# MicroRNAs as Guardians of the Prostate: Those Who Stand before Cancer. What Do We Really Know about the Role of microRNAs in Prostate Biology?

**DOI:** 10.3390/ijms21134796

**Published:** 2020-07-07

**Authors:** Thomas Andl, Kavya Ganapathy, Alexia Bossan, Ratna Chakrabarti

**Affiliations:** Burnett School of Biomedical Sciences, University of Central Florida, Orlando, FL 32816, USA; kganapathy@Knights.ucf.edu (K.G.); alexiabossan@knights.ucf.edu (A.B.)

**Keywords:** microRNAs, prostate biology, prostate cancer, androgen receptor signaling

## Abstract

Prostate cancer is the second leading cause of cancer-related deaths of men in the Western world. Despite recent advancement in genomics, transcriptomics and proteomics to understand prostate cancer biology and disease progression, castration resistant metastatic prostate cancer remains a major clinical challenge and often becomes incurable. MicroRNAs (miRNAs), about 22-nucleotide-long non-coding RNAs, are a group of regulatory molecules that mainly work through post-transcriptional gene silencing via translational repression. Expression analysis studies have revealed that miRNAs are aberrantly expressed in cancers and have been recognized as regulators of prostate cancer progression. In this critical review, we provide an analysis of reported miRNA functions and conflicting studies as they relate to expression levels of specific miRNAs and prostate cancer progression; oncogenic and/or tumor suppressor roles; androgen receptor signaling; epithelial plasticity; and the current status of diagnostic and therapeutic applications. This review focuses on select miRNAs, highly expressed in normal and cancer tissue, to emphasize the current obstacles faced in utilizing miRNA data for significant impacts on prostate cancer therapeutics.

## 1. Introduction

Accurate gene expression control is important for the formation, maintenance and repair of complex biological structures. One important regulatory element to ensure precise gene expression levels consists of small non-coding RNA molecules known as microRNAs. MicroRNAs (miRNAs) bind to the RNA-induced silencing complex (RISC) and are used to identify target messenger RNA (mRNA) transcripts. Once targeted by the miRNA-RISC using mainly a seed sequence region at the 5’ end of the miRNA for target recognition, these mRNAs are inhibited from being translated and are prone to degradation [1]. While this system may seem counterproductive, it is frequently used by cells to reduce gene expression noise, keep certain mRNAs in a specific expression range or confer robustness and stability to the gene expression pattern. Therefore, miRNAs fulfill critical functions in developmental processes, tissues and during tumorigenesis.

The exploration of miRNAs in prostate cancer is well represented in the scientific literature and several review articles have elaborated their function, e.g., [2]. However, many core questions remain unanswered and many key concepts correlating miRNA and prostate biology are misrepresented. In this review, we focus our attention on such key concepts and critical highly expressed miRNAs.

Before exploring the function of individual miRNAs in prostate biology, we want to explore a few topics that are important when evaluating miRNAs in prostate biology: 1., What miRNA expression data mean when considering that miRNAs may not all be in functional complexes that allow them to function as miRNAs; and 2., which cells express which miRNAs and how this influences the interpretation of miRNA expression data.

## 2. Implications of Low Molecular Weight (LMW)- and High Molecular Weight (HMW)-RISCs and the Conundrum of the Let-7 miRNA Family

A curious fact for consideration is that in many adult cells, “classical” miRNA function appears inactivated due to miRNAs being restricted to “inactive” low molecular weight (LMW) RISC complexes rather than active high molecular weight (HMW)-RISCs [3]. However, these LMW-RISC complexes likely still function as small interfering RNA (siRNA)-RISCs used to destroy foreign RNA.

A dichotomy of miRNAs, consisting of an “inactive” LMW form (with unclear but putative anti-viral functions) and an “active” HMW-form in growing cells, is reflected in the Dgrc8 knockout mouse prostate such that there is an absence of an obvious phenotype under normal conditions, but the presence of a strong phenotype upon tissue activation and tumorigenesis [4]. With these “inactive” RISCs in mind, we may require a fundamental re-interpretation of miRNA functions, especially of the most highly expressed miRNA families in prostate cancer such as the let-7 family of miRNAs. How changes in the expression of the let-7 family, and more importantly, changes in the RISC composition (LMW versus HMW) regulate prostate cancer is still poorly understood, exemplifying how little we really know. If let-7 is reduced by 43% in prostate cancer (one of the first reports in the literature for let-7a: [5]), but exists in inactive low-molecular weight RISCs in the normal prostate, how can let-7 be a tumor suppressor (e.g., [5])? Many studies did not find down-regulation of let-7 family members in prostate cancer versus normal prostate, e.g., [6]. Furthermore, it is not clear what the contributions of the other 12 let-7 family members are and whether they are reduced like let-7a. In one dataset (GSE80400), let-7a contributes only 21% to the let-7 family expression, which is consistent with findings in many cell lines (supplemental document S1 in [7]). Therefore, it remains ambiguous whether let-7 family activity or expression are reduced in prostate cancer. However, in vitro studies in prostate cancer cell lines showed suppression of let-7 increases proliferation and growth [8]. It makes sense to argue that even a weak reduction of the highly expressed let-7 family members can substantially affect control of target gene expression, but the identity of these target genes in prostate cancer is also still unclear.

Another obscure aspect of let-7 regulation by LIN28A suggests that we do not understand much about this miRNA family: LIN28A can suppress let-7 expression and is mentioned as a mediator of the “reduced” let-7 activity in prostate cancer [9]. Unfortunately, there is not much evidence that LIN28A or LIN28B are actually expressed in normal tissues or in prostate cancer cells (e.g., The Cancer Genome Atlas, TCGA, or other datasets). How can a gene whose expression is so highly restricted to ES cells and embryonal development [10] be able to suppress let-7 in adult tissues and cancer cells? There is a lack of evidence that LIN28 is induced or expressed in prostate cancer cells such as LNCaP, DU-145 or PC-3. Consequently, studies that present phenotypic changes in prostate cancer cells upon suppression of LIN28 [9,11] are hard to align with several experiments such as the ones documented in gene expression omnibus (GEO) datasets GSE85556, GSE71797, or GSE99381 that have shown a lack of expression of LIN28 in these cell lines.

Even for PC-3 cells, reports claim the expression of LIN28B [12] despite the absence of LIN28B mRNA based on RNAseq data (e.g., GEO dataset GSE80963). In an early report of LIN28’s role in tumorigenesis, the authors found few cell lines expressing LIN28, none of them prostate cancer cell lines (only 3.2% of all cell lines, i.e., 17 of 527 cell lines; [13]). Potential explanations for the insistence of LIN28 in the regulation of let-7 in prostate cancer are perhaps that LIN28A/B mRNAs are efficiently translated in prostate cancer cells into very stable proteins; or alternatively, that LIN28 antibodies show nonspecific binding and qRT-PCR Ct values were very high. For example, there is no indication of LIN28-mediated let-7 processing inhibition in the prostate cell line DU145 compared to cell lines that actually express LIN28 [7].

Lastly, prostate cancer does not exhibit frequent activation of oncogenes such as LIN28B through transposable elements as has been described for other cancers, e.g., lung cancer [14], further supporting the overall impression that LIN28 does not play a role in prostate cancer.

## 3. MiRNA Processing Machinery Genes Dicer and Dgcr8 in Prostate Biology and Their Surprising Loss of Function Phenotypes

These above examples exhibit the doubtful state of miRNA research in the field of prostate cancer. There also seems to be no clear miRNA signature that may distinguish normal prostate from cancer, at least when looking for consensus in the literature (“Any miRNA-based clinical screening still lacks a consensus signature to be applied in the routine assay, and needs further validation in an intended use population” [15]). In contrast to these ambiguous reports on individual miRNAs in prostate biology, the effect of ablation of Dicer suggests an important role of miRNAs in general in the prostate [16]. Interestingly, loss of *Dgcr8*, another essential RNA-binding protein to process miRNA primary transcripts, has little effect on the tissue [4]. Outside of the canonical miRNA processing, additional functions may explain the differences between *Dgcr8* and *Dicer* knockout mouse models. Dicer can function in the production of endogenous siRNAs and miRNAs, while Dgcr8 plays a role in the degradation of certain RNA species and small nucleolar RNA (snoRNA) processing [17,18]. Though there is hardly any difference among different knockout mouse models for other organs, the mouse prostate may represent an exception in that different phenotypes are manifested upon deletion [19]. Regardless of whether miRNAs are truly significant for prostate formation and maintenance, their requirement for mouse prostate tumorigenesis in a Pten knock out tumor model has been well documented [4].

## 4. A Major Confounding Factor in Interpreting miRNA Expression Data: Cell Type and the Lack of Comprehensive Characterization of Luminal Cells

Several obstacles have hindered significant progress in our comprehension of the role of individual miRNAs, specifically in prostate cancer. One of them is the lack of acknowledgment that prostate cancer is a “luminal cell disease”. Although the cell(s) of origin of prostate cancer(s) are still debated [20,21], most prostate cancers show progressive loss of basal cells and represent a luminal cell phenotype. This results in underrepresentation of basal cell-associated markers and genes (e.g., KRT5 or p63), including miRNAs in prostate cancer compared to normal prostate. However, this does not necessarily mean that basal cell miRNAs, such as miR-205, are tumor suppressors in prostate cancer [22] and are actually essential for mouse prostate tumorigenesis [23]. Alternatively, some reports indicate that miR-205 may be not entirely be restricted to basal cells and is also expressed in luminal cells and in prostate cancer cells [24]. Whether luminal miR-205 levels are relevant is unclear, but the stark differences in miR-205 levels between luminal and basal cells suggest that miR-205 may actually be a suppressor of the luminal cell phenotype [23] in line with the idea that miRNAs can suppress leaky transcripts that may interfere with correct differentiation and cell fate decision processes. Nevertheless, one of the major obstacles in prostate miRNA biology has been the lack of data on the miRNA expression pattern in luminal, basal, and non-epithelial cells; essentially, the lack of in situ hybridization (ISH) data or data of sorted cells. However, a steady stream of reliable ISH data and data on sorted cells will eventually clarify the cellular sources of individual miRNAs [23,24,25,26].

## 5. Characterizing miRNA Function through Rigorous Establishment of Relationship with Targets

MiRNAs may be considered to be equivalent to transcription factors in the post-transcriptional regulatory systems: they recognize short nucleotide sequences of seven nucleotides in length (seed sequence), work in larger protein complexes, and may work in a cell- and context-dependent manner [27]. Furthermore, like a transcription factor, a miRNA regulates a set of genes rather than individual genes. However, a major difference is that miRNAs generally do not act as master regulator switches of gene expression. Rather, miRNAs are fine-tuners and/or noise controllers [28,29]. In contrast, some data from Drosophila suggest that miRNAs can have one key target in some cell types during development which can entirely change the fate of the cell [30]. Whether this is a common theme or an outlier is not evident.

For miRNAs to fulfill their function during development or in “active” cells, they must influence their target genes. They can change target gene expression levels, repress leaky transcripts, and/or buffer the expression of the target mRNAs against unwanted fluctuations. Therefore, there are at least three types of targets: (a) mRNAs that are co-expressed and significantly regulated by fine-tuning of their expression (b) mRNAs co-expressed but present within a pool of excess target to temporarily soak up miRNAs [31] (c) mRNAs with mutually exclusive expression, in which case the miRNA may function as a suppressor of leaky transcripts. Recently, an excellent publication has eloquently summarized the topic on the pitfalls of miRNA research and target gene detection [32]. Here, we point out a few factors that may contribute to the existing ambiguity in miRNA research.

Analyzing the effects of a miRNA on its target mRNAs may predominantly involve 3’UTR (untranslated region) luciferase assays and physical association of the miRNA with the target mRNA. There is not one specific assay that by itself has enough authority to confirm this relationship with absolute certainty. Even one of the “gold standard” assays, the 3’UTR luciferase assay, is artificial and rather shows that a mRNA can be targeted by a specific miRNA without any indication whether this really may happen under physiological conditions [33]. Usually, the 3’UTR is overexpressed and reaches high levels which may or may not be physiological. When combined with overexpression of a miRNA, the abnormally high level of both can lead to artificial conditions. Relative quantity is important for the proper function. Similarly, assays showing interactions between RISCs and mRNAs or labeled miRNAs and mRNAs are also challenging, rendering the combination of multiple assays to strengthen the claim of a miRNA targeting an mRNA most appropriate.

It has become clear over the years that much of the data in miRNA literature have limited utility [32], and that many key ideas in miRNA biology, such as the competing endogenous RNA (ceRNA) hypothesis, may not be valid and plausible in their original implementation [34,35]. The above-outlined rules may help to sieve through the vast publication record on miRNAs in order to identify useful data in prostate miRNA biology. Databases are helpful tools which serve as an excellent starting point to find out what is known about miRNAs in a particular field of interest. For example, miRCancer is such a database that can provide a quick summary of miRNAs that are altered in a specific cancer as in, for example, prostate cancer.

The prostate miRNome has been studied intensively, but no real prostate-specific miRNAs have yet been identified. A broad comparison of 40 tissues by Liang et al. [36] showed that the prostate could be grouped together with tissues such as lactating breast, salivary glands, and seminal vesicles based on miRNA expression. Another similar microarray-based study with rat tissues resulted in the clustering of prostatic tissues with epididymis, testis, seminal vesicles, Harderian gland and mandibular gland [37]. Although both studies failed to identify any prostate-specific miRNAs, some datasets indicate that miR-143/145, miR-363, miR-375, miR-198 and/or miR-485 may be enriched in the prostate and may serve as useful markers of prostate cancer.

## 6. In the miRNA World, Quantity and Numbers Matter

The amount of miRNA in a cell directly influences target gene expression [38], a function summarized in [39], although exceptions to this rule may exist, as seen in an experimental model in Drosophila [40]. Another major caveat is that expression levels may not always reflect absolute activity and “functional availability” [41]. This becomes especially important when taking into consideration that most of the miRNAs present in cells in vivo might be in an inactive complex [3].

It has been estimated that depending on the cell type, cells can express around 10^5^ miRNA molecules and a similar amount of Argonaut [42,43]. In order to achieve repression by an individual miRNA, the miRNA level must be increased tremendously [38,39,40,41,42,43,44,45,46]. For instance, to achieve an additional 10% repression of a target gene, the miRNA level must increase 10-fold. However, one important aspect is that one mRNA can be targeted by multiple miRNAs. Therefore, an in vivo scenario could be different, hence it will be prudent to study the expression levels of multiple miRNAs that target a specific mRNA to understand the true effects of a dysregulated miRNA. However, these values from Kozomara et al. [40] may suffer from a limitation of using a luciferase reporter assay that pretends miRNAs are siRNAs. In their experiment, a perfectly complementary target sequence was used as one of the readouts for miRNA activity [40]. A more appropriate assay format would have used a more natural miRNA binding site as defined by a seed sequence rather than a completely complementary sequence.

Generally, too much focus is given to miRNAs with a high-fold difference in gene expression between two states, e.g., cancer and normal tissue. In many cases, these differentially expressed miRNAs show overall relatively little expression beyond the background level [40]. Since highly expressed miRNAs most likely occupy most RISCs, understanding their function is critical for the understanding of miRNA biology of the prostate. Furthermore, one of the important factors determining the ability of a miRNA to target an mRNA in a cell- and context-dependent manner is the miRNA expression level, which also can be an important factor in determining the level of non-seed/non-canonical targeting [47].

To identify which miRNAs fit the above-mentioned requirements, we performed a meta-analysis of several miRNA expression studies on prostate tissues and cell lines (Table 1). Using these criteria, only a handful of miRNAs remain that seem pertinent to therapeutic applications. Most of these miRNAs have a broad expression pattern and seem to represent a housekeeping core set of miRNAs which belongs to ancient miRNA families. These abundant miRNAs in the prostate can be regarded as “the guardians” in one of two ways: either they mediate post-transcriptional gene expression control, or they are involved in siRNA-mediated guard against foreign RNA similar to a bacterial Ago-mediated function, as alluded to by La Rocca et al. [3].

## 7. How to Evaluate miRNA Expression Data: A Cautionary Note

In the light of mounting evidence, it may be stated that miRNA activity may be low in adult tissues under homeostatic conditions [3] and expression levels measured by various methods may not reflect the actual miRNA activity [41]. These concerns raise valid questions as to what the miRNAs are really doing in normal adult tissues. To answer these questions, it is more challenging—yet important—to critically evaluate and interpret the plethora of data on miRNAs in prostate biology. If the majority of miRNAs were indeed “inactive” in normal tissues, it would suggest that normal prostate may not exhibit much miRNA activity after all; and just the activation of prostate epithelial cells during the tumorigenic process may induce the activity of most of these miRNAs. In that case, any highly expressed miRNA, irrespective of whether it is differentially expressed or not, is likely to exert an effect in the tumor but not in normal tissue. Consequently, almost in a paradoxical manner, these miRNAs could now be regarded as “oncomirs” since they are most likely activated in the tumorigenic process due to their recruitment to larger RISCs (HMW-RISCs). In other words, miRNAs may switch their function from an siRNA-like activity in low-molecular weight RISCs to their classical function in high molecular weight RISCs [3] during tumorigenesis. However, whether this interpretation will prevail or not is yet to be seen and requires thorough testing.

MiRNAs may function differently under homeostatic conditions as compared to activated or stressed conditions. The loss of Dicer, a key miRNA processing enzyme, does not cause any phenotypic effects in vivo if the tissue is not “activated” and/or highly proliferative. Several examples from the field in skin biology may be enlightening here. Tissue or cell activation, e.g., through induction of hair growth, results in the manifestation of Dicer knock-out (KO) phenotypes [19]. On the other hand, in vitro keratinocytes and most other cell types seem to be incapable of growing without Dicer and become senescent. Since in vitro growth generally represents an activated state which for keratinocytes is often compared to a woundlike phenotype, Dicer KO manifests very clearly under these conditions. Furthermore, the overexpression of miRNAs in rodent skin generally has little effect unless the hair growth cycle is activated or the skin is under stress. A last indication that miRNAs may function differently under homeostatic conditions stems from the observation that the vast majority of miRNA knockout animals hardly show any phenotypic changes unless they are placed under stress conditions [53,54,55]. This concept can also be applied to the prostate. One of the most abundant miRNAs in basal cells of the prostate, miR-205, can be eliminated in mouse prostates without any consequences [23]. However, challenging the tissue in the absence of miR-205, e.g., by promoting prostate carcinogenesis, will produce a profound phenotype of cancer suppression [23].

With this background information and ideas on miRNA function, evolution and regulatory potential, we would like to shed light on the role of a selected group of miRNAs in prostate biology and prostate cancer. As in the case of all rules, there are exceptions that do not fit into our categories and they have been covered by other review articles [56,57,58,59]. Here, we will primarily focus on abundantly expressed prostatic miRNAs with dramatic expression changes following the idea that quantity matters [60]. Furthermore, we narrowed down our selection of miRNAs based on their potential roles in establishing resistance towards androgen deprivation therapy and their role in metastasis. A colossal study by Song et al. compared dozens of studies on miRNA expression in prostate cancer and serves as a solid platform to narrow down relevant miRNAs [15]. Their meta-analysis shows that few miRNAs are consistently altered in prostate cancer. In Table 1, we summarize our meta-analysis that indicates that a few microRNAs can be put in an “oncomiR” category, i.e., they are overexpressed in prostate cancer. These miRNAs include miR-375, miR-148/152, miR-182/183/96, miR-22, and miR-141/200/429. On the other hand, some miRNAs superficially fit into a “tumor suppressor” category such as miR-143/145, miR-125, and miR-23/24/27. Our review will explore whether such categorizations are meaningful at the current state of prostate miRNA research.

All of these miRNAs are abundantly expressed. However, no other miRNAs are expressed as highly in prostate as the miR-143/145 cluster, and no other miRNA cluster has been as misinterpreted as miR-143/145.

## 8. MiR-143/145: Highly Expressed vs. Highly Important

### 8.1. The miR-143/145 Cluster Is a Stromal Cell Marker

The expression of the miR-143/145 cluster has been studied intensively in mice using a beta-galactosidase (lacZ) reporter. The analysis of beta-galactosidase (lacZ) activity showed highly restricted expression in smooth muscle cells [61]. Since the prostate is rich in smooth muscle tissue, it is not surprising that this tissue has high levels of miR-143/145 and these miRNAs compromise the major miRNAs in prostate tissues. During prostate cancer progression, the stroma gets reorganized [62] and levels of the putative smooth muscle marker miR-143/145 become down-regulated [63]. Nevertheless, it is still one of the most highly expressed miRNAs in prostate cancer tissue when compared to other miRNAs (Table 1).

Surprisingly, Larne et al. describe a highly enriched expression of these miRNAs in basal cells of the prostate and not in smooth muscle cells [63], while Boettger identifies the lacZ signal in the prostate stroma and not in the epithelium [61]. Additionally, Zedan et al., have conclusively shown that the vast majority of miR-143/145 are derived from non-epithelial cells [25]. In addition, Wach et al. [64] could not detect such a distinct miR-143/145 expression in prostate tissues as described by Larne et al. [63]. Additionally, in situ hybridizations (ISH) of mouse ureters and intestines of control and miR-143/145 knock-out mice, as well as murine lung, human colon, and human bladder cancer, confirmed expression in smooth muscle cells [65,66,67,68]. ISH and qRT-PCR of human gastric tumors have shown the expression of this miRNA in stromal cells but not tumor cells. [69]. In vitro analysis using human cell lines also seem to show miR-143/145 cluster expression at significant levels exclusively in fibroblasts ([69]; and Human Genome browser GRCh37/hg19 miR-143HG gene RNAseq from nine cell lines from ENCODE; most recently: [70]). Furthermore, miR-143/145 are highly expressed in the PNF08 cell line, which is derived from normal prostate fibroblasts but not prostate cancer cell lines [71].

The only other indication that the miR-143/145 cluster may be expressed in prostate basal cells stems from the studies performed by Rane et al. [72]. The strength of their analysis lies in the sorting of cells with basal cell features from prostate tissues. However, their analysis only indicates differential expression in the basal cell population of the prostate gland but not its absolute expression level.

One recent in situ hybridization study specifically explored miR-143/145 in more detail using tissue microarrays of prostate tissues and large numbers of cases and controls [26]. The study confirmed the preferential expression of both miR-143 and miR-145 in stromal cells. The authors could not detect miR-145 in cancer cells, but they describe cytoplasmic expression of miR-143 mainly in metastatic lesions. However, they were able to visualize miR-143 in the transitional zone of the prostate in epithelial cells. It is unclear how these two miRNAs from the same cluster can exhibit such diverse expression patterns, or how these data fit with the data presented above that have shown exclusive expression of both miRNAs in non-epithelial cells. Furthermore, the location of the in situ hybridization (ISH) signal is interesting and seems to diverge from some previous studies as well: all the miR-145 signal is in the nucleus. However, upon closer inspection of Zedan et al.’s data [25], their ISH signals appear primarily nuclear as well. Overall, the study by Eckstein et al. [26] showed surprisingly frequent expression of miRNAs in the nucleus and even in the nucleoli of cells, although nucleoli are generally a rare location for miRNA expression [73]. If these data are correct, then the question arises: how do these miRNAs properly function in translation control if present primarily in nuclei?

There is now ample evidence that mature miRNAs are located and active in the nucleus and that they are utilized to regulate the activity of promoters, enhancers, mRNAs, and long non-coding RNAs [74]. However, a nuclear function has not been explored for any of the miRNAs discussed in this review article and the focus of all these studies has been on a cytoplasmic function of miRNAs, despite the fact that several of these miRNAs are primarily expressed in the nucleus.

Overall, the claim that the miR-143/145 cluster is a tumor suppressor in prostate cancer is made less plausible by the fact that miR-145 is either a basal cell marker or most likely a pure stromal cell marker and prostate cancer is a “luminal disease” with loss of basal cell characteristics. This issue has recently been outlined by Witwer and Halushka in their review article stating that “*microRNAs are expressed by cells, not tissues*”. This illustrates that our knowledge base in the miRNA field rests too often on ambiguous grounds [32].

### 8.2. The Fallacy of Overexpressing a Stromal microRNA in Prostate Cancer Cells

If this miR143/145 miRNA cluster is expressed in smooth muscle cells, and neither in normal nor in malignant prostate epithelial cells, then overexpression or manipulation of these miRNAs in the epithelial cells will probably add little to our understanding of prostate biology [75]. Nevertheless, assuming the miR-143/145 cluster is relevant for prostate biology, the knowledge of the target genes of these miRNAs could help to unravel their function. Surprisingly, the targetomes of these two “tumor-suppressive” miRNAs have not yet been explored in a comprehensive manner, especially not in prostate stromal cells [60]. Most publications focus on *one* target gene to explore the phenotypic changes mediated by these miRNAs. However, data from whole genome transcriptome analyses on miR-143/145 knockout tissue identified a few good candidates such as IGFBP5, PDGFRA, CBFB, and PPP3CA, especially when combined with results compiled through TarBase.

### 8.3. For Few miRNAs in Addition to miR143/145 the Cell Type of Expression Is Known

Similar to the case of the miR143/145 cluster, other miRNAs have been studied in more detail by ISH. Two studies stand out here for evaluating the expression of several miRNAs using ISH [70,76]. For example, miR-30c was down-regulated during cancer progression, showing the lowest expression in metastatic disease. However, this study [76] also remains overall superficial when it comes to the question of basal versus luminal cell differentiation. An example highlighting the importance of the spatial resolution of gene expression comes from Melbø-Jørgensen et al. [77]. The authors show that miR-21 can actually be frequently expressed in the prostate tumor stroma rather than the tumor cells and miR-21 is one of the most highly expressed miRNAs in prostate cancer. The study by Kumar et al. [70], however, shows mainly stromal and not epithelial expression of miR-1, miR-143, and both stromal and epithelial expression of miR-21. Interestingly, studies by Iscaife et al. showed anti-metastatic effects of miR-145 (generally co-expressed in stromal cells with miR-143) upon systemic delivery of miR-145 [78]. Although it is unclear if the effects are mediated through replenishment of the stromal pool, it is important to pay attention to the cell type specific expression of miRNAs under study for understanding prostate cancer biology.

**Figure 1:**
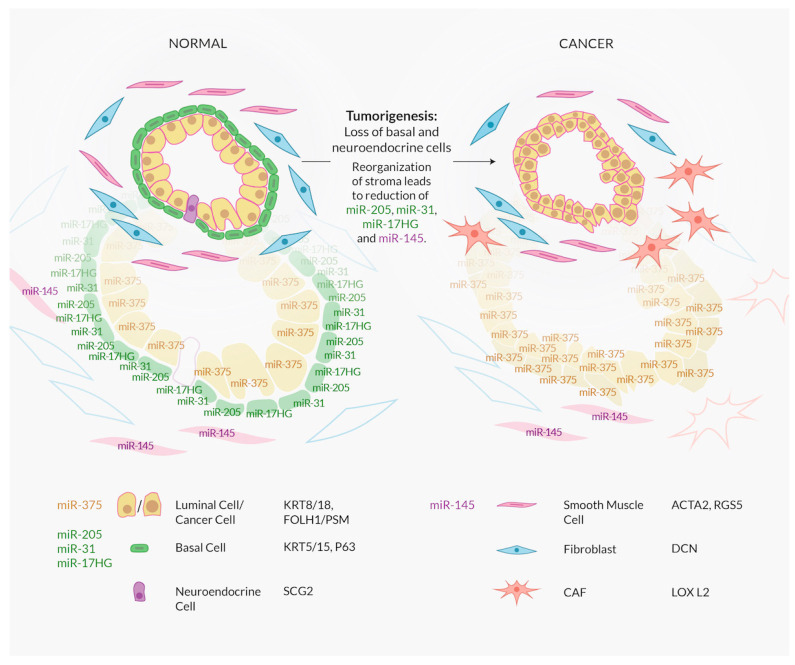
Overview of the changes in tissue organization that underlie some changes in miRNA and protein expression. Reduction in the expression of miR-205, miR-31, miR-145, and the miR-17-92 cluster are frequently observed in prostate cancer studies. These changes can be explained by the loss of basal cells (miR-205, miR-31, and miR-17HG), reorganization of the stroma (reduction in miR-145) and overexpression of miR-375 in cancerous luminal cells. Similar changes can be observed in the expression of mRNAs coding for basal cell markers such as KRT5, KRT14, or p63, while luminal markers KRT8/18 and Folate Hydrolase 1 (FOHL1/PSM) are frequently overexpressed in prostate cancer. It is still unclear, due to a lack of comprehensive miRNA in situ hybridization (ISH) data, which miRNA changes in prostate cancer are mainly due to changes in tissue cell composition. A recent study on normal mouse prostate suggest that few miRNAs show substantial differences in expression with the exception of miR-205, miR-17, miR126a (basal) and miR-375 and miR-148a (luminal) [23].

In Figure 1, we summarize the little information about the cell-type specific expression of miRNAs we have in the prostate biology field. Based on the available data, miR-145 is an almost exclusively stromal miRNA, while miR-205 is a basal cell marker and miR-375 appears to be the only luminal cell-associated miRNA.

## 9. MiR-375: An Indeterminant Oncogene, a Mirage of a Tumor Suppressor Gene

### 9.1. miR-375 a Luminal Epithelial Phenotype Gatekeeper?

According to the TCGA prostate cancer RNAseq dataset, miR-375 is highly expressed in prostate cancer and elevated compared to normal prostate, which is confirmed by in situ hybridization experiments [26]. As a repeated occurrence with many miRNAs, available data show that miR-375 defies simple classification as tumor suppressor miR or oncomiR. MiRNAs seem to work in a highly context-dependent, cell-type specific manner [27] and can show opposite effects within a cell lineage as it is the case with miR-375 [79]. Costa-Pinheiro et al. and Selth et al. [80] have shown miR-375 varies tremendously between prostate cancer cell lines and its inhibition can alter the phenotype and transcriptome in PC-3 and 22Rv1 cells. They also pointed out a potential link between miR-375 and androgen receptor (AR) levels, which had been previously detected by Chu et al. [81]. Their work further supports that miR-375 may be used to differentiate between subgroups of prostate cancer. This relates especially to prostate cancer and cancer cell lines that may have more mesenchymal features (lower miR-375) compared to ones with more epithelial features (higher miR-375) since overall “miR-375 is elevated in prostate cancer and associated with an epithelial phenotype” [80]. Therefore, miR-375 may be called the keeper of the epithelial phenotype in prostate cancer especially luminal epithelial cell features. miR-375 is one of the few luminally enriched miRNAs (at least 10-fold) in the dataset by Fan et al. [23]. MiR-375 may function as a gatekeeper and it stands before the transition to more aggressive and more mesenchymal forms of prostate cancer (PCa). In that sense, miR-375 could be regarded a tumor suppressor.

### 9.2. The Molecular Mechanisms How miR-375 May Function as A Gatekeeper Are Unclear

Several studies have explored the function of miR-375. An elegant study by Choi et al. integrated miR-375 into a “regulatory axis” with the Capicua transcriptional repressor (CIC) at its heart [82]. The study determined that CIC is a marker of both luminal and basal cells with its expression reduced in prostate cancer. However, confirmation through the Human Protein Atlas is absent [83], which only supports expression of CIC in basal cells. If CIC is preferentially expressed in basal cells and its progressive loss in prostate cancer is due to the loss of basal cell type features, then this merely supports the notion that prostate cancer is a “luminal disease” with minimal change in CIC expression in cancerous cells compared to normal luminal cells. This finding indicates that the basal/luminal dichotomy does not only pose problems for the interpretation of miRNA data but also for the expression of protein-coding genes. However, the authors showed that the combined action of miRNAs miR-93, -106b, and -375 could down-regulate CIC in vitro and promote growth of PC-3 cells.

A similar study using PC-3 cells showed inhibition of invasion and viability by miR-375 [79] while Choi et al. [82] suggest a different outcome in combination with miR-93 and -106b. Several recent studies have focused on other miR-375 target genes. Two studies focused on Yes-Associated Protein 1 (YAP1) as a target gene [80,84] while CBX7 has been deduced as a potential mediator of miR-375 activity from comprehensive Ago immunoprecipitation experiments [85]. Interestingly, neither YAP1 nor CIC were identified as miR-375 target genes in the screening carried out by Pickl et al. [85] and neither CIC nor CBX7 were predicted target genes based on TargetScan [86]. However, re-analysis of the Pickl et al. screening data involving TargetScan-predicted target genes indicates that these genes are enriched for Hippo/YAP1 signaling. This suggests that miR-375 may indeed be a regulator of Hippo signaling in prostate cancer cells alongside the fact that YAP1 expression is reduced in prostate cancer (Human Protein Atlas [83,87]). miR-375 may also coordinate with miR-200 to control “epithelial plasticity in prostate cancer” [80,88]. However, all these interpretations rest on the premise of miR-375 presence in cytoplasmic RISC complexes, but as inexplicably reported in Eckstein et al. [26], most miR-375 is associated with nucleoli in human prostate tissue samples. However, whether this association of miR-375 with nucleoli is an artifact or not remains to be seen since some miR-375 ISH probes tend to cross-hybridize with rRNA in nucleoli [89]. Therefore, a clear picture of what the actual function of miR-375 is in prostate cancer and beyond has still to emerge.

### 9.3. No Conclusive Data on miR-375 Categorization as OncomiR or Tumor Suppressor

As indicated above, data on miR-375 are often inconclusive as to whether this miRNA is an oncomiR or a tumor suppressor. For example, miR-375 is one of the top down-regulated miRNAs in oral cancer, playing a tumor suppressor role by targeting CIP2A which stabilizes the MYC oncoprotein [90]. MiR-375 is a potential prognostic biomarker in oral cancer, however, in contrast to prostate cancer (PCa) where up-regulation is associated with cancer, in oral cancer miR-375 down-regulation is prognostic [91]. Other studies show that miR-375 can function as a tumor suppressor in gastric cancer by targeting PDK1, YWHAZ and Janus kinase 2 and in hepatocellular carcinoma by targeting YAP1 and MTDH [92].

Some of the confusion on whether miR-375 is tumor suppressive or ongogenic may result simply from technical problems. For example, massive un-physiological overexpression of miR-375 may “always” result in a growth-suppressive phenotype in vitro in prostate cancer cells or for that matter any cancer cell (e.g., oral cancer). To explore this issue in a more unbiased fashion and shed light on the unsatisfactory conclusions about miR-375’s role in cancer, we selected ten of the most recent publications [93,94,95,96,97,98,99,100,101,102] and ten of the most cited publications [103,104,105,106,107,108,109,110,111,112] that deal with miR-375 and determined the pro- or anti-growth outcomes of its overexpression or inhibition in these studies. Overexpression using a miR-375 mimic produced growth inhibition and/or induction of apoptosis in 16 of the 17 studies, while miR-375 inhibition had either no effect or mild pro-growth effects in 7 of 10 studies. The starkest example for the lack of any major effect of miR-375 inhibition comes from Merkel cell carcinoma (MCC) cell lines that express high levels of miR-375. Even almost complete inhibition of miR-375 had almost no effect on the cells in vitro [99]. Only three studies (two on breast cancer cell lines MDA-MB231 and MCF7 and one on miR375 knockout in mouse pancreas) indicate an oncogenic or at least pro-growth effect of miR-375 [100,101,102,103,104,105,106,107,108,109,110,111].

Comparing all these non-prostate cancer studies with the four studies exploring the role of miR-375 in prostate cancer [79,80,81,82,83,84,85] may suggest a different role of this miRNA dependent of cell type. Although rather inconclusive (two studies show growth promotion and two show growth inhibition of PC-3 cells by miR-375 overexpression!), there is the possibility that miR-375 may indeed be an oncogene in prostate cancer (PCa) initially, and perhaps upon progression may be a hurdle to a more aggressive state. Alternatively, the majority of these studies on miR-375 are an example of the replication crisis in science [113].

Beyond experimental troubles of un-physiological expression changes, other issues could explain the troubling inconsistencies in the current miR-375 publication record. A frequently overlooked aspect of human tumors is their tumor cell heterogeneity. Not all tumor cells proliferate, nor do all tumor cells have the same differentiation status. Some cells, for example, may be exposed to hypoxia. At invasive fronts of tumors, cells often undergo partial epithelial-mesenchymal transition and may down-regulate miR-375 that support this transition. A better and more detailed analysis of ISH data on PCa samples are required to shape the current inconclusive and paradoxical data into a coherent model of how miR-375 may regulate PCa biology.

Currently, the most probable conclusion from studies on miR-375 is that it is neither a powerful oncogene nor a strong tumor suppressor gene. The 24 above-mentioned publications on miR-375 too often contradict each other. For example, two studies argue that miR-375 suppresses growth in PC-3 cells and two studies claim the opposite. Furthermore, the sobering lack of a phenotype from loss of miR-375 expression in MCC cell lines suggests that miR-375 may have functions outside the tumor cells, as suggested by Fan et al. [99] and supported by studies in prostate cancer [114]. An exosomal function of miR-375 must be explored in more detail, as well as the idea that exosomes loaded with sufficient amounts of miR-375 can be transferred to adjacent cells to mediate an effect in the recipient cells.

## 10. MiR-22: Possibly an Androgen-Regulated Tumor Suppressor miRNA: Few Data, Big Ideas

miR-22 is highly expressed in the prostate and prostate cancer cells (Table 1) and shows a trend for up-regulation in PCa. However, the functional significance of this up-regulation remains obscure. One tool to get an idea of the functional significance of a miRNA is to evaluate the phenotype of knockout mice, especially under stress conditions. miR-22 affects the fertility of mice, although the mechanism underlying the reduced litter sizes and survival of miR-22 knockout mice is unknown. Heart failure, kidney problems and altered hair growth have also been observed in these mice [115]. Notably, heart tissue has the highest expression of miR-22. Under stress conditions, miR-22 is required in the kidney to induce TGF-β superfamily mediated fibroblast activation, fibrosis, and collagen production. However, it is unclear which target genes mediate miR-22 function indicating an obstacle as no clear picture has emerged to better understand miR-22.

The rule of thumb, although not undisputed [27,47], has been that miRNAs generally have target genes in a non-cell-type specific manner [116]. However, such a dataset is not readily available for miR-22. Without such knowledge it seems injudicious to declare miR-22 as a therapeutic target in cancer, especially with unclear functions as a tumor promoter or suppressor [117].

### 10.1. MiR-22 and Its Relationship with Androgen Receptor (AR) Signaling

MiR-22 is one of the few miRNAs regulated by androgen receptor activity [118,119,120,121]. This makes it an interesting candidate for mediating effects of androgens in normal and cancer cells, especially as it is down-regulated in hormone-refractory prostate cancer and has a role in tumor progression. This idea is derived from the analysis of an in vitro progression model using M2182 and M12 cells, derived from the p69 cell line. Comparing these three related cell lines shows a three-fold increase of miR-22 expression in the more aggressive cells [122]. These data are strengthened by the study of Budd et al. with their use of laser capture microscopy (LCM), and therefore topical information, to compare the expression of miR-22 in normal prostate epithelium with tumor cells. Again, miR-22 was up-regulated about three-fold in the tumor cells. Zehner’s group is among the few that have frequently applied LCM to overcome one of the great limitations in miRNA expression studies [123]: the lack of spatial information and analysis regarding the cell type and its corresponding miRNA, and vice versa.

The initial association of miR-22 with progenitor cell populations in the breast epithelium may be helpful. These progenitor cells are also high in miR-205 and miR-31, two other potential basal or myoepithelial cell markers of exocrine glands, such as the breast and the prostate (see Figure 1, GEO dataset GSE120716 from [124] which shows a specific enrichment of precursors of miR-205, -31 and -17 in basal cells), along with miR-22-2p [125]. However, there is no indication in the Henry et al. dataset [124] that miR-22 is actually enriched in basal cells.

### 10.2. Confusing Experiments Putting miRNAs in Regulatory Axes Which May Not Exist

Several studies have looked into the mechanisms of how miR-22 may regulate prostate cancer cell behavior. For example, Dhar et al. [126] confirmed that E-cadherin is a good candidate for a human miR-22 target gene, but this study also highlights the fallacy of the assays used to determine regulation. Their hypothesis is that MTA1, an epigenetic regulator, induces miR-22 expression and miR-22 in turn suppresses E-cadherin expression, which thereby increases aggressiveness. The problem emerges here when the authors switch to a mouse model inherently lacking explanation on how miR-22 may regulate E-cadherin, since no miR-22 target sites have been predicted for mouse E-cadherin (reference TargetScan, Miranda, and miRDB) and the human E-cadherin site is poorly conserved. Therefore, the correlation observed in mice between MTA1/miR-22/E-cadherin is unlikely to be based on miR-22 targeting E-cadherin directly but rather by other mechanisms. Consequently, whether the hypothesis is true for humans is questionable since the “same” outcome is reported in the mouse and the human system: MTA1 induces miR-22 which in turn seems to down-regulate E-cadherin by an unknown pathway. The fact that the overexpression of MTA1/miR-22 seems to mimic human tissue/cell outcomes despite a lack of a miR-22 binding site in mouse E-cadherin could indicate that either the 3’UTR assays are—again—unreliable indicators for in vivo regulation or there are data missing for full explanation of the authors’ hypothesis. This further suggests that mouse models must be carefully evaluated for miRNA research when building a case for a specific miRNA-target mRNA relationship. Another striking example for problems translating findings in real human tumors into mouse models is the complete lack of regulation of the miR-141/200/429 family by ERG in mouse prostate due to a lack of ERG binding sites in the miR-141/200/429 promoter while these miRNAs are regulated by ERG in human prostate cells [127].

The miR-22 case highlights our desire to tell unsubstantiated stories about complex processes involving miRNAs. In other publications, an AR/MYC/miR-22/PHF8 axis has been proposed [128]. This raises the question whether the MTA1/miR-22/E-cadherin axis is truly operational in human cells or some of the interpretations are tremendously oversimplifying miRNA biology. The strength of the AR/MYC/miR-22/PHF8 axis rest on the fact that PHF8 is a top predicted target gene of miR-22 and the miR-22 target site in the PHF8 3’UTR is extremely well conserved in mammals. However, the axis suggests that miR-22 is down-regulated in prostate cancer. Although some studies support this idea [71,118], other studies suggest up-regulation and an oncogenic activity of miR-22 [123,129]. Eventually, a thorough analysis of the miR-22 target genes in prostate cells will help to determine whether this miRNA is generally a mediator of anti-apoptotic [130,131,132,133] or pro-apoptotic signals, which would make it an attractive therapeutic target [118,134,135]. In any case, in contrast to miR-143/145, miR-22 is a genuine prostate epithelial cell miRNA with functions relevant to prostate cancer.

## 11. MiR-148a: A Preferential oncomiR in Tumor Cells Amidst Proposed AR Signaling

Still following the core principle of prioritizing highly expressed miRNAs, a study on prostate metastasis identified miR-148a as a dominant miRNA in their defined setting. The next-generation sequencing of a prostate metastasis model suggests that miR-148a is a major prostatic miRNA [136]. A quick crosscheck of TCGA miRNA sequencing data of human prostate cancers reveals that miR-148 is indeed one of the top human miRNAs in prostate cancer tissues (Table 1; https://tcga-data.nci.nih.gov/tcga/). Studies by Szczyrba et al. further showed that miR-148 is up-regulated in prostate cancer [71]. The simplest explanation of miR-148a overexpression in prostate cancer is its preferential expression in normal luminal cells as has been determined from fluorescence-activated cell sorting (FACS) of purified mouse prostate cells: miR-148a-3p is about four-fold enriched in luminal cells compared to basal cells [23].

However, most of the evidence that miR-148 is oncogenic is circumstantial as follows: its expression is down-regulated when prostate cancer cells are exposed to anti-proliferative and chemo-preventive drugs [137]; it is up-regulated in prostate cancer compared to the normal prostate [49]; high grade tumors seem to have reduced levels of miR-148 [138]; miR-148a is associated with growth of castration-resistant prostate cancer and its overexpression in LNCaP cells promotes proliferation in this cell line [52] and shows regulation by androgens [139,140]; in a large screen of miRNAs associated with oncogenic functions, miR-148 was one of the few that showed positive effects on several prostate cancer cell lines [141].

In contrast, a CpG island close to miR-148a is methylated indicating transcriptional suppression in prostate cancer cell lines, with azacytidine treatment leading to restoration of its expression. However, such methylation was relatively rare in human prostate cancer samples in vivo. Furthermore, in hormone-refractory prostate cancer cell lines such as PC-3 and DU145, miR-148a has growth-suppressive functions. In addition, miR-148a is reduced in docetaxel-resistant variants of prostate cancer cell lines including PC-3 and DU145. MiR-148a is also down-regulated in prostate cancer with risk of biochemical failure [142]. Again, this indicates context-dependent functions of miRNAs. miR-148a is no exception and it can be declared a tumor suppressor or an oncogene depending on the cell type and stage of tumor progression [143]. A better definition of the target genes of miR-148a will help to decipher the production of differential phenotypes in different cell types. Only one study has performed an unbiased survey of target genes [144] and identified “cell-to-cell signaling and interaction” as a major association of miR-148a target genes. ITGA5 was one of the key target genes and its modulation could explain some of the phenotypes that were produced by miR-148a in breast cancer cells. The interesting thing about miR-148 is that changes in its expression may mark critical steps in tumor progression, especially its down-regulation in advanced castration-resistant prostate cancer (CRPC) patients. It may therefore be a marker of a catastrophic shift in prostate cancer biology from manageable tumor to the currently incurable CRPC.

## 12. The miR-182/-183/-96 Cluster: Overexpression Associated with Zinc Homeostasis, Angiogenesis, Hypoxia and Androgen Receptor Signaling

The miR-183/-182/-96 cluster is frequently overexpressed in prostate cancer (e.g., [145]) and may be associated with high grade cancers [146]. The miRNAs of the cluster are generally pro-tumorigenic and increase proliferation in prostate cancer cell lines. Two factors have been identified as potential drivers of their overexpression: androgen receptor signaling and hypoxia [145]. In most AR-positive cell lines, the expression of the cluster is elevated compared to AR-negative ones. Additionally, several characteristics of prostate cancer may be regulated by this cluster. For example, Zinc is enriched in normal human prostate but reduced in prostate cancer. High Zinc levels are associated with another characteristic of human prostate: high citrate levels. Therefore, the mechanisms that regulate Zinc uptake and transport are important for prostate biology. There is evidence that the miR-183/-182/-96 cluster can inhibit Zinc uptake in prostate cancer cells and thereby mimic the stark decrease of Zinc in prostate cancer tissues [147,148].

Since the cluster represents three different miRNA families with different seed sequences, one can expect to have a multitude of candidate target genes that regulate proliferation, migration, angiogenesis, and Zinc uptake. However, the Nonn laboratory has shown that the large miR-183/-182/-96 cluster may not be perfectly co-regulated but has specific regulatory elements that may explain differential expression of cluster miRNAs that result in 10–100 fold elevated levels of miR-182 compared to miR-183 [147]. Whether these elements also mediate androgen-responsiveness of miR-182 remains to be shown [145]. In mouse prostate cancer cell lines miR-96 can be regulated by TGF-β [149] but it is unclear whether the same is true for the entire miR-183 cluster and for human prostate cancer cell lines. Siu et al. determined that miR-96 expression correlates with TGF-β signaling and TGF-β target gene expression in a set of human prostate cancer samples [149]. Interestingly, the tumor-promoting effects of miR-96 seemed either dependent on the p53 or AR signaling status of the mouse and human prostate cancer cell lines. Further studies will be needed to detangle the roles of miR-183/-182/-96 in prostate cancer and how each individual miRNA is regulated and contributes to cancer growth.

## 13. MiR-141/-200/-429: Controlling Epithelial Plasticity and Metastasis

A miRNA family with a seemingly straight-forward role in prostate cancer is the up-regulated five-member strong miR-141/-200/-429 family, suggesting an oncomiR role. However, the devil is in the detail. A major function attributed to this miRNA family is stabilization of the epithelial phenotype and suppression of epithelial-mesenchymal transition (EMT), a process associated with invasion and metastasis in many cancers. Some of the earliest reports on this miRNA family in prostate cancer indicated that their members are indeed involved in mediating the EMT phenotype in PC-3 cells by regulating the mesenchymal factors ZEB1, ZEB2 and SNAIL1 [150,151]. This perplexing inhibition of EMT while aiding proliferation may be explained by differential expression in subpopulations within the tumor. Prostate cancer is an epithelial tumor and therefore will express considerable levels of miR141/-200/-429 family members (see Table 1; [15]) which may promote proliferation of these epithelial cells. However, cancer populations are heterogeneous and subpopulation of cancer cells that drive invasion and spread of the cancer may benefit from reduced levels of miR-141/-200/-429. Indeed, low level expression of the family is associated with cells displaying features of prostate cancer stem cells [152]. CD44, a marker for prostate cancer stem cells, is reportedly suppressed by miR-141/200/429, thus prostate cancer samples and cell lines with high CD44 feature very low levels of miR-141/-200/-429. As expected by Liu et al., miR-141 promoted epithelial and suppressed mesenchymal features which seemed in part to be mediated in part by “genes associated with cytoskeleton remodeling and cell motility, including Rho GTPases”. Consequently, this gene expression pattern mediated by miR-141 led to suppression of metastasis in a xenograft mouse model.

In prostate cancer, the experiments by Liu et al. [152] and others have roughly defined a 90-gene target signature for miR-141/-200/-429 which shows enrichment for genes involved in the regulation of the actin cytoskeleton. This well-defined targetome will prove invaluable for Liu et al. and future experiments into the paradox that miR-141 suppresses proliferation yet induces “a partial EMT phenotype that allows a great proliferative capability of epithelial cells and the morphological plasticity of mesenchymal cells”. Nevertheless, there still remain inconsistencies difficult to explain: why do groups report that overexpression of miR-141/-200/-429 is oncogenic [153,154,155], while the overwhelming majority contradicts this idea (e.g., [127,155,156,157,158,159,160])? There also might be differences and variations between the same prostate cancer cell line in various laboratories accounting for striking differences in miRNA gene expression (Table 1; see comparison of miR-141/-200/-429 expression from various published manuscript figures). In some publications, PC-3 has hardly any difference to other prostate cancer cell lines. Perhaps these differences can, in extreme cases, lead to opposite results when studying specific miRNAs.

Another cautionary note can be made here in regard to PC-3: it is astonishing that three studies have shown that overexpression of at least four unrelated miRNAs, miR-141 [152], miR-375 [80], miR-126 [161], and the miR17HG cluster [162] virtually result in the same phenotype, i.e., the induction of a more epithelial phenotype and re-expression of E-cadherin. Whether these findings indicate that studies on PC-3 and miRNAs must be re-evaluated, or that PC-3 is extremely sensitive to changes in miRNA expression levels, is unclear. An alternative view is that a major hub of miRNA activity in prostate cancer centers on EMT and E-cadherin regulation since not only do miR-141, miR-375, and miR17HG function as guardians of the epithelial phenotype, but miR-22 also targets the same pathway. Mir-22, however, may repress E-cadherin (at least in humans) and favor a mesenchymal phenotype.

Recently, another twist has been added to the role of the miR-200 family: miR-200 can regulate the RNA-binding protein QKI, which in part affects mRNA splicing. Pillman et al. [88] interrogated datasets to identify miR-200 target genes and, as expected, found mediators of epithelial-mesenchymal transition such as ZEB1 and ZEB2 to be high on the list. Surprisingly, the second highest ranking gene of their analysis was QKI, which itself is modulated during EMT [163]. The authors confirmed that miR-200 as well as miR-375 can target and regulate QKI. In their main cell culture system of breast cancer cell lines, QKI had a profound effect on mRNA splicing, suggesting that miR-200/miR-375/QKI control alternative splicing during EMT. The same result could be confirmed in LNCaP cells, indicating that this QKI function is broadly regulated by the putative epithelial caretakers, miR-200 and miR-375. Taken together, regulation of miR-200 of QKI appears to have a profound effect on alternative splicing, especially of genes associated with the actin cytoskeleton and in extension of the phenotypic changes associated with EMT.

## 14. The Link Between High Grade Prostate Cancer, Neuroendocrine Differentiation, and miRNAs: miR-30

One of the current key targets of prostate cancer therapy is the androgen receptor (AR). Failure to respond to AR-centered therapy and development of castration-resistant prostate cancer (CRPC) is associated with diverse molecular mechanisms applied by prostate cancer cells [164]. One of these mechanisms involves the switching of tumor cells to a neuroendocrine differentiation and/or growth independent of AR signaling [165]. This mode may be accompanied by a specific set of miRNAs that characterize neuroendocrine cells and tumors.

As indicated in our discussion about miR-375, several miRNAs associated with neuroendocrine cells are highly expressed in prostate cancer tissues and cell lines. For example, the miR-182/-183/-96 cluster and miR-9 fall into this category. However, in prostate cancer miR-182 seems to be associated with AR signaling and not with neuroendocrine differentiation [145]. One study showed a strong increase in miR-9 and miR-30 expression in tumors with a high Gleason score [166], suggesting that miR-30 may also accompany the neuroendocrine switch. However, as common with prostate miRNAs, contrasting reports indicate a reduction of miR-30 in prostate cancer [167], which may occur in tumors with low “neuroendocrine” program activation. Fitting into this scheme is the fact that miR-30 can impair AR activity [168]. Furthermore, some studies suggest that miR-30 family members can inhibit prostate cancer cell proliferation while others show the opposite (e.g., [39] versus [169]). This controversy is well documented and discussed in Lin et al. [169]. However, recent data from the same group on miR-30d has brought some insights into the targetome of the miR-30 family in prostate cancer [169]. A 36-target gene signature has been identified for neuroendocrine cancer, which is enriched in negative regulators of transcription.

**Figure 2:**
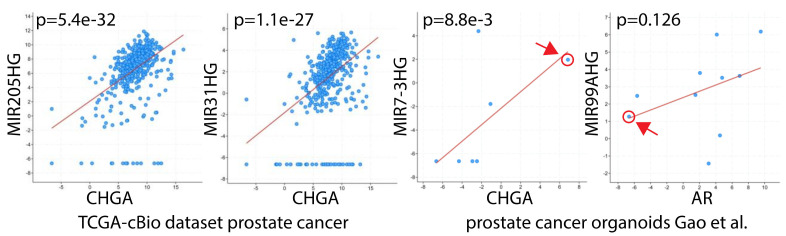
Relationship between neuroendocrine-like differentiation (CHGA^+^, miR-7^+^) in response to androgen suppression therapy. TCGA data and data from Gao et al. [171] were analyzed using the cBioPortal (https://www.cbioportal.org) using the co-expression tool [172]. Expression values are in log2. P-values are for the Spearman test. The red circles and arrows depict a prostate cancer organoid with neuroendocrine characteristics (CHGA^+^, miR-7^+^, AR^−^, miR99a^low^, plus a lack of cytokeratin expression [171]) derived from a “prostate cancer with treatment-induced neuroendocrine differentiation”. Collectively, these data suggest that true and complete neuroendocrine differentiation is rare in prostate cancer.

Despite the emerging importance of neuroendocrine-like features in prostate cancer in response to therapy, astonishingly little is known about this “switch”. A recent study has put some “meat” on the bare bones of this theory of a neuroendocrine switch. Ostano et al. have compared PCa with classic adenocarcinoma features with those that have signs of neuroendocrine differentiation [170]. Surprisingly, the miRNAs associated with this switch appear to be basal cell miRNAs such as miR-31. We could confirm this with a simple search of the TCGA dataset using the cBioPortal for miRNA associated with the neuroendocrine marker Chromogranin A (CHGA) in PCa samples (Figure 2). Ostano et al. and our review of the TCGA data suggest that a true and complete neuroendocrine differentiation switch appears to be rare in response to anti-androgen treatment.

## 15. MiR-378: A Link Between Tumor Cachexia and Tumor Progression

Although miR-378 is not one of the most abundant miRNAs in the prostate, it can still be robustly detected and has been implicated in tumor biology and metabolism. miR-378 is not an ancient miRNA and is restricted to placental mammals. Accordingly, its expression is generally not as high in most tissues as is generally the case for ancient miRNAs. This may appear to be a strange statement since evolutionary emergence and gene expression levels do not necessarily correlate, but in the cancer miRNA world, dominant miRNAs in the transcriptome are typically ancient and highly conserved [173].

Members of the miR-378 family show specific expression in epithelial cells of the prostate since comparing cultivated “normal” epithelial prostate cells or LNCaP cells with stromal fibroblasts or myofibroblasts show exclusive expression of miR-378b, -378f, -378g and -378i in the epithelial cells similar to members of the miR-200 family, miR-203, the miR-183/-182/-96 cluster, and miR-205 [174] (supplemental Table S5). On the other hand, in this dataset miR-199, miR-143/-145, miR-409, miR-411, miR-127, and miR-100 mainly showed expression in fibroblasts.

MiR-378 is down-regulated in many types of cancer, including prostate cancer. A plethora of potential target genes has been investigated, validated, and used to explain its role in tumor progression. However, loss of miR-378a in mice has implications in mitochondrial energy control, especially with miR-378-3p being the major mature miRNA of the miR-378 family in this species [175]. This function of miR-378 is surprisingly reflected in its differential expression upon exercise and changes in body fat content in humans [176,177]. Unfortunately, according to the miRbase, the miR-378 family has four members in mice, three of which have identical seed sequences. In humans, the situation is even more complex, with eleven miR-378 genes, nine of which have the same seed sequences. This diversity makes it difficult to evaluate the contribution of individual family members.

Increased serum levels of miR-378* (miR-378a-5p) are associated with prostate cancer [178]. However, loss of miR-378 in tumor samples has been linked to more aggressive prostate cancer [179]. It will be interesting to see how these contradictory findings of increased serum miR-378 levels and decreased tumor levels of the same miRNA may be explained. One possible link is tumor cachexia [180]. It is possible that the increased serum levels of miR-378a-5p do not indicate changes in the tumor itself, but changes in the tissues responsible for fat storage and lipid metabolism during tumor progression. Since miR-378 is up-regulated in fat tissues of patients with tumor cachexia, one could speculate that fat tissue is the source for the increased serum levels of members of the miR-378 miRNA family [180]. So, how could a reduction of miR-378 alter prostate cancer cell metabolism? Valentino et al. suggest that miR-378 targets enzymes that mediate the transport of fatty acids into mitochondria, thereby decreasing the usage of fatty acids for energy production. They identified that miR-378 could suppress the expression of Carnitine O-acetyltransferase (CRAT), an important enzyme in the shuttle of fatty acids into mitochondria. Therefore, reduced expression of miR-378 could contribute to altered fatty acid usage in prostate cancer cells [181].

## 16. MiRNA Relationships with Androgen Receptor (AR) Signaling

A key therapeutic target in prostate cancer is Androgen receptor (AR) signaling. Accordingly, substantial interest has been dedicated to the regulation of this signaling pathway by miRNAs. Androgen Deprivation therapy (ADT) using anti-androgens such as Enzalutamide and Abiraterone is commonly used to target and inhibit AR signaling for therapeutic purposes in prostate cancer. Therefore, understanding the altered expression of miRNAs that target AR and are AR-regulated can help to understand the development of resistance to therapy. Since a recent review article has dealt with the subject comprehensively [182], we would only like to highlight a few aspects of the relationship between miRNAs and AR.

Several candidate miRNAs have been determined to target AR directly [52,168,182,183]. As we have mentioned earlier, miR-30 family members are some of the most prominent in prostate cancer cells that can directly target AR.

Most metastatic prostate cancers are androgen independent with constitutively active AR signaling due to different adaptations such as AR amplification, mutations in the ligand binding domain leading to promiscuous activation, making it more difficult to treat. Some miRNAs are involved in the earlier stages of prostate cancer progression such as miR-346 which is highly expressed in cancer tissues compared to normal prostate whereas some other miRNAs such as miRs-197 and -361-3p show high expression specifically in Enzalutamide-treated CRPC xenografts and may be required to maintain prostate cancer progression in the later stages upon anti-androgen therapy [184]. Furthermore, tumor suppressor miRNAs such as Let-7 and miR-30b, -30d, -200 family or oncogenic miRNAs such as miR-141, -125b, -21 and -32, target the AR and play a role in the progression of prostate cancer to the more aggressive, metastatic castration-resistant stage. Some of these miRNAs have been explored more in detail in previous chapters of this review and using this information to sieve through the abundance of miRNA data could lead to the identification of potential therapeutic miRNAs to treat CRPC [185].

On the other hand, AR signaling influences the expression of a set of miRNAs including miR-148, -182, -22, -29, and -21 [182]. Interestingly, in CRPC characterized by reduced AR signaling, one can observe the paradoxical increased expression of AR-induced miRNAs such as miR-182/183 and the reduction of AR-inhibited miRNAs such as miR-99 and miR-100. This could be due to alternative mechanisms of regulation of these miRNAs that are largely independent of AR signaling and perhaps more related to changes in cell type composition of the cancer tissue. However, at least one group of miRNAs, the miR-23, -24, -27 clusters, shows the expected regulation: miR-27a can be induced by AR signaling [186,187] and miR-23b, -24, and -27a are reduced in CRPC [188,189]. Expression of the miRNAs of the two miR-23, -24, -27 clusters are generally reduced in prostate cancer and the clusters are associated with tumor suppressive activities [190]. Unfortunately, little is known about the function of these miRNA clusters and how they may mediate changes in AR signaling activity. More studies are required to elucidate whether the miR-23, -24, -27 clusters have diagnostic or therapeutic implications.

## 17. Serum/Plasma miRNA Levels and Relation to Diagnosis and Prognosis

In recent years, ideas have emerged regarding the use of miRNAs in serum, plasma or urine as potential tools in the diagnosis of cancer, to predict the aggression level and to monitor the efficacy of treatment. The poor reproducibility of this approach was highlighted in a study of breast cancer [191]. However, the dire need for better non-invasive tests to determine prostate cancer is highlighted in meta-analyses of current procedures such as prostate specific antigen (PSA) tests and digital rectal examination, which provide little evidence that PSA tests have an impact on prostate cancer mortality [192]. However, some correlations were observed between circulating miRNAs and serum PSA (e.g., [193]), suggesting that miRNAs may possess diagnostic value.

An important factor to consider in using miRNAs present in body fluids as source for diagnostic tests, is the original source of these miRNAs. There is a good correlation between the miRNAs in the urine and the expression of miRNAs in kidneys and prostate [194]. The same is true for serum, plasma, and feces as starting material. This means that the emergence of a “new” tissue such a prostate tumor should have a measurable impact on the miRNAs found in urine. A recent summary on urine miRNAs and their value for diagnosis of prostate cancer suggest that more comprehensive studies are needed with more standardized procedures [195]. Consequently, only a handful of miRNAs emerged from this metanalysis and could be candidates for further investigation. One of them being miR-375.

A study by Mihelich et al. [196] also re-evaluated many potential serum indicator miRNAs for prostate cancer and confirmed the loss of specific miRNAs in aggressive and recurrent disease states, advocating for use in prognostication. Interestingly and strangely, all the miRNAs they identified were downregulated in the serum of more advanced or recurrent prostate cancer. Additionally, not all 14 miRNAs showed the same trend in other studies. Furthermore, a different study identified miRNAs that were primarily overrepresented in serum of prostate cancer patients compared to benign prostatic hyperplasia, BPH [197]. This study by Haldrup et al. confirmed miR-375 as a biomarker for prostate cancer, which had been identified previously [178,197,198,199,200,201]. Therefore, miR-375 may currently be the best miRNA candidate for a novel serum (and urine) biomarker for prostate cancer, especially if one recalls that miR-375 is enriched in prostate tissues. One of the few studies using RNAseq and qRT-PCR assays showed that low miR-375 in serum is associated with patient survival. Although the vast majority of studies show elevated miR-375 levels in serum plasma of prostate cancer patients, the frailty of theses serum and plasma assays is highlighted by a contradictory report claiming that miR-375 is reduced or unaltered in the plasma of cancer patients [202,203]. The authors identified that once larger cohorts are analyzed, additional blood tests based on miRNAs could be manufactured that would complement PSA tests [204,205] or could be combined with the PSA test [206]. A recent study supports the potential for miR-375 to become a valuable marker for prostate cancer management and could be used to improve active surveillance of patients [207]. A detailed study on the presence of “free” serum miRNAs versus miRNAs in extracellular vesicles again supports that miR-375 may be a reliable marker when using total plasma [208]. In this study only few other miRNAs such as miR-200 and miR-21 had diagnostic power when analyzed within the extracellular vesicle compartment.

Recently, a study by Urabe et al. evaluated how extracellular vesicles (EV) could themselves be regulated by miRNAs and how EV production could increase in prostate cancer cells by altering miRNA expression [209]. The authors identified miR-26a as a potential regulator of EV secretion and concluded that the observed reduction in miR-26a in prostate cancer progression may cause an increase in EV secretion and consequently in prostate cancer aggressiveness. They also could show that in addition to miR-26a, three protein coding genes regulated EV secretion, i.e., SHC4, PFDN4, and CHORDC1, and that all these three genes can be direct targets of miR-26a. Therefore, miR-26a may negatively regulate EV production by suppressing SHC4, PFDN4, and CHORDC1. Although it is a convincing study, there are some concerns in regard to its significance: miR-26a levels are reduced only mildly in prostate cancer and some studies suggest it is up-regulated [210]. It is unclear whether such a mild suppression can impact EV production. No miR-26 inhibition was performed to show that lowering its levels can indeed significantly increase EVs. The miR-26 targetome also differs significantly from an earlier study which did not identify any of the above mentioned genes [211]. Nevertheless, these findings suggest that miRNAs in body fluids may eventually be successful diagnostic tools because tumor cells may produce increased amounts of secreted miRNAs.

An important factor to consider for diagnostic use of miRNAs in prostate cancer is the striking similarities to miRNA changes and expression patterns observed in bladder cancer. For example, the miR-143/145 cluster has also been implicated to be significantly down-regulated in bladder cancer [212]. Additionally, Ghorbanmehr et al. [213] have shown that miR-21, miR-141-3p and miR-205-5p are up-regulated in the urine of bladder cancer and prostate cancer patients with a potential as diagnostic and prognostic marker for both cancers.

## 18. Therapeutic Applications on the Horizon

MiRNAs can be viewed as attractive targets for cancer therapy [214,215]. Since most miRNAs are relatively reduced in their expression in prostate cancer, the therapeutic approach would most likely involve re-expression of certain miRNAs in prostate cancer tissues. A notable exception is miR-21, the putative prostate cancer-associated fibroblast marker whose suppression may prove beneficial for patients. Although several RNAi-based drugs have advanced in clinical trials [216], with several companies investing in RNAi- and miRNA-related drug development, it is difficult to evaluate them in competition with the major, currently trending cancer therapy strategies, including targeted therapies and immunotherapies.

The first RNA-based drug, Fomivirsen, was approved more than 17 years ago by the U.S. Food and Drug Administration and still remains on the market, although its commercial success is limited [217]. Reagents specifically targeting miRNAs as well as miRNA replacement drugs such as miravirsen (SPC3649; [218]), MRX34 [219], or TargomiRs [220] are in clinical trials. However, recent setbacks clearly indicate a long path ahead in making RNAi and miRNA-based therapies an achievable goal. TargomiRs, a miR-15/16 and nano cell-based therapy that attempts to re-establish the activity of these miRNAs in lung cancer cells has resulted in a case report indicating that such miRNA-based therapies may work ([221]; https://clinicaltrials.gov/ct2/show/NCT02369198) but the MRX34 trial has ended due to toxicity, poor results, and five immune-related serious adverse events (https://clinicaltrials.gov/ct2/show/NCT01829971?term=mrx34&rank=2; [222]). Prostate cancer research has been included in these efforts to use RNAi as therapeutic agents, although it is still at the preclinical stage [223]. McNamara et al. have described an approach to specifically target cells expressing FOHL1 with siRNAs against survival genes such as PLK1 and BCL2. In vivo mouse models have demonstrated that the growth of a FOHL1-positive tumor cell line (LNCaP) could be inhibited with this novel drug.

Another route to modulate miRNA activity and expression has been explored recently using miR-21 as an example [224]. The authors re-designed the RNA-binding protein RBFOX to specifically recognize the precursor of miR-21 and initiate its degradation. Such an approach has not been explored intensively and may provide a fascinating alternative to current approaches exploiting miRNAs in prostate cancer [225].

Notwithstanding, many hurdles must be overcome to make miRNA- and RNAi-based therapies a competitive alternative to the other major efforts in cancer therapy [226]. Initial steps have already been made and impressive advances in delivery tools for small RNAs have greatly complemented them [227,228,229].

## 19. Conclusions

Despite a vast publication record on the role of miRNAs in the prostate, few consistencies as to the function of these small RNAs in prostate cancer have emerged. The majority of differentially expressed miRNAs in prostate cancer are progressively down-regulated in their expression during tumor progression, as has been reviewed previously [59]. Whether miRNA function is down-regulated accordingly has become a critical question in miRNA biology that has yet to be addressed, and should be a focus of future studies [3]. Only once the major players in prostate epithelial and stromal cells have been established can a better understanding of the role of miRNAs emerge for therapeutic and diagnostic exploitation.

Even with more than a decade of intensive miRNA research, many issues in the field are still controversial, e.g., the competitive endogenous RNA hypothesis [230], non-seed sequence mediated binding of about a third of all Argonaute-associated mRNAs [47,86,231,232,233], or the role of extracellular vesicle transfer of miRNAs [234] or whether 84% of all miRNAs truly exist, and how many are maintained during evolution [235,236]. These unsolved ambiguities of miRNA biology also impact prostate research since the perspective of the role of miRNAs seems to change with different authors’ acknowledgment of disputed issues in miRNA biology [32]. Although current knowledge has yielded satisfactory solutions for some of the core questions of miRNA biology [233], many are still debated and have given rise to further questions, which have to be addressed before miRNAs and their regulatory circuits can be exploited for prostate cancer therapy [237].

Contradictions and inconsistencies in the prostate miRNA publication record are not trivial or victimless. The canon of miRNA publications serves as the basis for new ideas, projects, grant applications, and clinical trials. Therefore, a critical review of the current standing of miRNA research in prostate biology is vital.

## Figures and Tables

**Table 1:** Overview of the most highly expressed microRNAs (miRNAs) in normal prostate, prostate cancer (PCa) tissues and prostate cancer cell lines. MiRNAs were ranked from 1 to 10 in each of the datasets that were used to generate this table. If a miRNA was not ranked in the top10 of a dataset, then this is indicated by a “-”. ^$^: miRNA family or cluster (bold highly conserved, ancient common to proto- and deuterostoma). Striking differences for example between ^5–8^ experiments all on LNCaP: miR-378, miR-30, miR-21 are some of the highest in ^8^ but are far less expressed in the other three experiments indicating differences in culture methods or differences due to LNCaP subclones that may have evolved during prolonged cultivation. It remains to be seen whether the miRNAs that are differentially expressed between the LNCaP “clones” are relevant or not. A “-” means that in this particular experiment, the miRNA was not in the top 10 expressed group of miRNAs. For example, in the last “in vitro” column for “Clip RNAseq” for five prostate cancer (PCa) cell lines, “6/-/-/8/-” in the miR-125 column means that members of the miR-125 were expressed in the top 10 in LNCaP and DU145. In the other three cell lines, at least 10 other miRNAs were more highly expressed than miR-125 family members. Each experiment included in the table is represented either by a number or rank or a “-” indicating the expression level/rank in each experiment with “-” indicating that the miRNA was not ranked in the top 10. N: normal, PCa: prostate cancer. ^1^: examples from the TCGA miRNA RNAseq data collection on prostate cancer (https://cancergenome.nih.gov). ^2^: [48]. ^3^: [49]. ^4^: GSE71081. ^5^: [50]. ^6^: [51] ^7^: GSE71336. ^8^: GSE66035. ^9^: GSE40026. ^10^: [52].

	In Vivo Top 10 Expressed			In Vitro Top 10 Expressed				
microRNA ^$^	TCGA ^1^ Rank	N ^2,3^	PCa ^2,3^	RWPE ^4^	LNCaP ^5,6,7,8^	Microarray ^9^ (RWPE/ LNCaP/ PC3/ DU145/ Stromal)	Clip RNAseq ^10^ (LNCaP/ LAPC4/ 22Rv1/ DU145/ PC3)	Altered in Cancer
miR143/145	1	1/1	1/1	-	-/-/-/-	-/-/-/-/-	-/-/-/-/-	down
**miR125/99/100/10**	**2**	**2/5**	**2/3**	-	-/**3**/-/-	-/**1/10**/-/-	**6**/-/-/**8**/-	**down**
**miR375**	**3**	-/-	**4**/-	-	-/-/-/-	-/-/-/-/-	-/-/-/-/-	**up**
miR148/152	4	-/-	9/-	-	-/-/2/5	-/-/-/-/-	3/2/1/6/-	up
miR21	5	-/10	-/10	1	-/4/6/1	8/-/7/2/-	10/-/-/3/3	
miR30	6	-/-	-/-	-	-/-/-/3	-/-/-/10/-	9/-/-/-/-	
**miR182/183/96**	**7**	-/-	-/-	**7**	-/-/**5/6**	-/-/-/-/-	**2/1/4/4/1**	**up**
**miR22**	**8**	**8/8**	-/**9**	**4**	-/-/**4**/-	**9**/-/-/**8/6**	**5/8**/-/**7/6**	**up**
**let7**	**9**	**3/3**	**3/4**	**3**	**1/1/1/2**	**3/2/1/4/1**	**1/3/2/5/2**	
**miR141/200/429**	**10**	-/**9**	-/**7**	-	-/**6/10/8**	**10/10**/-/-/-	**7**/-/-/-/-	**up**
Mir-23/24/27	-	5/2	7/2	5	-/-/9/-	1/-/3/1/2	-/-/-/2/7	down
miR-26	-	6/6	6/6	9	-/-/8/-	-/7/-/-/-	-/-/9/-/-	
**miR-191**	-	**7**/-	**5**/-	**10**	**2**/-/**3**/-	-/-/-/-/-	-/-/-/-/-	
**miR-15/16**	-	-/**7**	-/**8**	-	**5/8**/-/-	**7**/-/-/**9**/-	-/-/**8/9**/-	
**miR-29**	-	-/**4**	-/**5**	-	**6**/-/-/-	-/-/**9/7/7**	-/-/-/-/**8**	

## Abbreviations

3’UTR	3’untranslated region of the mRNA
Ago	argonaute
AR	androgen receptor
BPH	benign prostatic hyperplasia
ceRNA	competing endogenous RNA
CRPC	castration-resistant prostate cancer
Ct	threshold cycle
dsRNA	double-stranded RNA
EMT	epithelial-mesenchymal transition
ENCODE	Encyclopedia of DNA Elements
FDA	Food and Drug Administration
GEO	Gene Expression Omnibus
HMW	high molecular weight
ISH	in situ hybridization
KO	Knock-out
lacZ	β-galactosidase
LCM	laser capture microscopy
LMW	low molecular weight
miRNA	microRNA
Pca	prostate cancer
PSA	prostate specific antigen
qRT-PCR	quantitative reverse transcription-polymerase chain reaction
RISC	RNA-induced silencing complex
RNAi	RNA interference
RNAseq	high-thoughput RNA sequencing
siRNA	small interfering RNA
TCGA	The Cancer Genome Atlas
TGF-β	tumor growth factor beta
